# Erwachsenen- und Weiterbildung unter Pandemiebedingungen. Herausforderungen und Perspektiven

**DOI:** 10.1007/s40955-021-00197-0

**Published:** 2021-11-15

**Authors:** Kerstin Hoenig, Gabriele Molzberger

**Affiliations:** 1 Deutsches Institut für Erwachsenenbildung – Leibniz-Zentrum für Lebenslanges Lernen, Bonn, Deutschland; 2 Bergische Universität Wuppertal, Wuppertal, Deutschland

Noch ein weiteres Themenheft zur Corona-Pandemie?, mögen sich manche fragen. Nach rund zwei Jahren ist vieles über „die Pandemie“ gesagt, gedeutet und geschrieben worden. Eine Reihe von Themenheften relevanter Zeitschriften und weitere Diskursorgane haben für eine Befassung und wissenschaftliche Auseinandersetzung mit der Pandemie und ihren Folgen Raum geboten. Auch Forschende im Feld der Erwachsenen- und Weiterbildung haben die Pandemie – nach berechtigter anfänglicher Zurückhaltung – zum Gegenstand von Untersuchungen gemacht.

In der Zusammenschau unterschiedlicher Publikationen ist die pandemische Krise, gesehen als Wendepunkt, als Chance oder als Entscheidungsmoment, ein wiederkehrendes Thema. In manchen Beiträgen der öffentlichen Debatten und des fachwissenschaftlichen Diskurses über die Pandemie wurde auch das Verhältnis von Wissenschaft und Öffentlichkeit zum Gegenstand. Die Erwachsenen- und Weiterbildung ist davon in ihrem wissenschaftlichen Selbstverständnis sowie als Forschungs- und Handlungsfeld in besonderer Weise betroffen. Popularisierung von Wissen – in einem wohlverstandenen, nicht abwertenden Sinne gemeint – bleibt im Zusammenspiel mit anderen Disziplinen eine ihrer zentralen Aufgaben.

Ausgangspunkt dieser Ausgabe der ZfW ist, dass sich die Bedingungen und Möglichkeiten der Bildung Erwachsener in ihren sozialen, kulturellen und beruflichen Kontexten mit der Pandemie verändert haben. Auswirkungen zeigen sich auf der Ebene von gesellschaftlichen Rahmenbedingungen, organisationalen Voraussetzungen und Formaten sowie lernender Aneignungen in den verschiedenen Institutionalformen von Erwachsenen- und Weiterbildung. Auch das Spektrum von Themen und Inhalten hat sich verschoben, weil indirekt oder direkt durch die Pandemie ausgelöste Anforderungen und Bedarfe an Kenntnissen und Fähigkeiten sich nicht nur im Bereich der beruflichen Weiterbildung niederschlagen.

Inwieweit die oben angesprochene Rede von der „Krise“ bzw. „Disruption“ oder ob die Sprachbilder von der Pandemie als „Brennglas“ bzw. „Katalysator“ zutreffend sind, wird sich erst mit einigem zeitlichen Abstand beurteilen lassen. Noch lässt sich nicht diagnostizieren oder antizipieren, in welchen revidierten Vorstellungen über Gesellschaft, soziales Zusammenleben sowie über die Aufgabe und Funktion von Bildung sich die Pandemieerfahrung im Zusammenspiel mit anderen Krisenwahrnehmungen der Gegenwart niederschlägt. Öffentlich wurde und wird die frühkindliche und schulische Bildung mit Intensität diskutiert. Man wird die Relevanz der Frage, wie Kinder und Jugendliche der „Generation Corona“ von der Pandemie geprägt sind, auch kaum bestreiten mögen. Daneben stellt sich die Frage, auf welche Weise die Bildung Erwachsener von der Pandemie betroffen ist. Welche Lebensverläufe und biografischen Transitionen werden verzögert, gebrochen, in ihrer Richtung umgelenkt oder beschleunigt – dauerhaft oder zeitweilig? Welche alten Privilegien der Bildungsteilhabe werden verstärkt, welche neuen Benachteiligungen erwachsen? Welche neuen Anforderungen entstehen für das Bildungspersonal? Wie reagieren die Weiterbildungsanbieter kurz- und mittelfristig auf veränderte Bedingungen und Bedarfe? Und vor allem, welche Rolle spielt Erwachsenen- und Weiterbildung dabei genau? Einordnungen zu ihrer Funktion und Aufgabe im gesellschaftlichen Gefüge müssen fortlaufend erfolgen.

Zu einigen Aspekten dieser Fragen lassen sich erste Trends ausmachen und liegen erste Erkenntnisse vor. Die in diesem Heft versammelten Studien zur Erfassung des äußerst diversifizierten Weiterbildungssektors knüpfen teilweise an bekannte, etablierte Untersuchungen an und zeigen Veränderungen auf.

In ihrem Artikel „COVID-19 und Weiterbildung – Überblick zu Forschungsbefunden und Desiderate“ stellen *Anika Denninger* und *Bernd Käpplinger* die bisherige Forschung im Feld der Erwachsenen- und Weiterbildung zur Pandemie mit dem Ziel vor, die bisherigen Studien zu systematisieren und Forschungslücken zu identifizieren. Dabei beinhaltet der Beitrag im Gegensatz zu üblichen Forschungsüberblicken auch Studien, die noch im Entstehen sind oder zu denen keine Veröffentlichungen vorliegen. Dies trägt der Schnelllebigkeit der aktuellen Entwicklungen Rechnung. Denninger und Käpplinger stellen fest, dass sich die bisherige Forschung insbesondere auf Weiterbildungsanbieter sowie Lehrende fokussiert, wohingegen die Folgen der Pandemie für die Teilnehmenden bisher unerforscht sind. Darüber hinaus identifizieren sie sieben thematische Schwerpunkte für die zukünftige Forschung und liefern damit neue Impulse.

*Susanne Wißhack und Sabine Hochholdinger* befassen sich in ihrem Beitrag mit der Frage: „Wie erleben und bewältigen Lehrende der berufsbezogenen Weiterbildung Folgen der Corona-Pandemie?“ Die Fragestellung wird mithilfe einer qualitativen Textanalyse der Webbeiträge von 22 Lehrenden der beruflichen Weiterbildung untersucht. Dabei dient das GRETA-Kompetenzmodell als theoretische Grundlage, auf dessen Basis drei Forschungsfragen abgeleitet werden: Auswirkungen der Pandemie auf die berufliche Situation, sich daraus ergebende Anforderungen an pädagogisch-psychologische Kompetenzen sowie notwendige Kompetenzen im Vereich der Selbstregulation und Bewältigung. Die Befunde bieten Implikationen sowohl für Lehrende und Weiterbildungsanbieter zum Umgang mit Krisen und der Erweiterung des digitalen Angebots als auch für theorie- und evidenzbasierte Forschung zu Kompetenzen von Lehrkräften insbesondere im Hinblick auf digitale Kompetenzen.

Dem informellen berufsbezogenen Lernen während der Corona-Pandemie widmet sich der Beitrag von *Corinna Kleinert, Gundula Zoch, Basha Vicari und Martin Ehlert*. Auf Basis einer Sondererhebung des Nationalen Bildungspanels (NEPS) untersucht die Autorengruppe, ob das informelle Online-Lernen als Reaktion auf die Pandemie im Frühjahr und Sommer 2020 zugenommen hat und welche sozioökonomischen und beruflichen Faktoren mit der Nutzung digitaler Lernangebote in Zusammenhang stehen. Mithilfe von logistischen Regressionen wird dabei die Wahrscheinlichkeit der Nutzung digitaler informeller Lernangebote – beispielsweise Lern-Apps, Videotutorials, Internetforen oder Wikis – in der letzten Erhebungswelle vor der Pandemie sowie in einer Sondererhebungswelle im Mai und Juni 2020 geschätzt. Dabei zeigt sich, dass die Krise den „digital divide“ zumindest in der Frühphase der Pandemie eher verschärft als reduziert hat. Eine zunehmende Nutzung digitaler Lernangebote erfolgte disproportional häufig bei höher Gebildeten und bei Befragten, die im Home-Office arbeiteten.

Der vierte Themenbeitrag von *Johannes Christ, Andreas Martin und Stefan Koscheck,* „Zur Anpassungsfähigkeit von Weiterbildungsanbietern in der Corona-Pandemie“, geht der Frage nach, welche Arten von Weiterbildungsanbietern in der Krise besonders flexibel von Präsenzangeboten auf digitale Formate umstellen konnten. Dabei lauten die Hypothesen, dass sich erstens das Maß der digitalen Vorerfahrung begünstigend auf die Transformation von Präsenz- in Online-Formate auswirkt und zweitens kommerzielle Anbieter innovationsfreudiger als nichtkommerzielle Anbieter sind und daher eher kurzfristig auf Online-Formate umstellen können. Die Autoren schätzen auf Basis der wbmonitor-Daten den kausalen Effekt der digitalen Vorerfahrung mithilfe eines Dose-Response-Modells und den Effekt kommerzieller Anbieter über ein Difference-in-Differences-Verfahren. Beide Hypothesen können durch die rigorosen Analysen nicht bestätigt werden. Der Artikel gibt damit Hinweise dafür, dass die kurzfristige Reaktion auf die Krise komplexeren Mustern folgt und weitere theoretische und empirische Arbeit nötig ist.

Insgesamt decken die vier Beiträge des Themenheftes ein breites Spektrum ab, sowohl aus inhaltlicher als auch methodischer Perspektive. So werden Lehrende, Weiterbildungsanbieter sowie Teilnehmende in den Blick genommen. Dabei einigt die Beiträge der Fokus auf die Bedeutung digitaler Lernangebote in der Pandemie. Wie auch der Überblick von Käpplinger zeigt, ist die Forschung zur Corona-Pandemie – trotz eines wachsenden Korpus publizierter Studien – noch keinesfalls abgeschlossen.

Im Vergleich zu früheren Publikationen fällt auf, dass die theoretische und methodische Fundierung der Beiträge zugenommen hat. Die hier vorgestellten empirischen Beiträge analysieren die Effekte der Pandemie auf Basis bestehender Theorien und entwickeln diese teilweise weiter. Dabei wurde komplexes Datenmaterial aufwändig aufbereitet und unter Rückgriff auf fortgeschrittene forschungsmethodische Designs ausgewertet. Frühere empirische Untersuchungen mussten dagegen zwecks schneller Veröffentlichung oft auf rein explorative, deskriptive Analysen zurückgreifen.

Allerdings ist festzustellen, dass diese inhaltliche Tiefe einen Preis hat. Die Beiträge in diesem Heft beziehen sich mit Ausnahme des Literaturüberblicks in ihrer Datenbasis weiterhin auf die Frühphase der Pandemie im Frühjahr und Sommer 2020. Hier geraten die Bedarfe der Praxis nach schnellen Antworten auf drängende Fragen und Probleme in der Krise in Konflikt mit dem Bedarf der Wissenschaft nach gründlicher theoretischer und empirischer Arbeit.

Auch bleiben trotz der inhaltlichen Vielfalt offensichtliche Lücken bestehen. Keiner der hier veröffentlichten Beiträge befasst sich mit der Makroebene des Weiterbildungsystems oder arbeitet regional oder international vergleichend. Auch auf der Meso- und Mikroebene bleiben zahlreiche Fragen offen, so etwa die Frage nach den Auswirkungen der Pandemie auf die Relation der Teilbereiche in der Erwachsenen- und Weiterbildung oder auf der Ebene der Teilnehmenden die Identifikation besonderer (auch gruppenspezifischer) Weiterbildungsbedarfe. Ebenfalls auffällig ist, dass uns keine Einreichungen erreicht haben, die sich an eine Prognose wagen, welche Folgen die Pandemie für die Zukunft der Erwachsenen- und Weiterbildung haben wird. Das Thema muss und wird die Weiterbildungsforschung – auch nach diesem Themenheft – weiter begleiten, ohne die Relevanz anderer Forschungsfragen zu schmälern. Das Herausgebergremium der ZfW hat diesem Umstand Rechnung getragen und ruft im Call for Papers für das kommende Heft – neben weiteren Themen – weiterhin zur Einreichung von Beiträgen zur Corona-Pandemie auf.

Fünf Forschungsbeiträge finden sich im Forum dieses Heftes. *Stefanie Hoffmann, Veronika Thalhammer, Aiga von Hippel und Bernhard Schmidt-Hertha* befassen sich mit Ursachen und Bedingungen von abgebrochenen Weiterbildungsteilnahmen, sogenannten„Drop-outs“. Auf Basis einer empirischen Studie schlagen sie eine Typologie von Nicht-Passungen bei Drop-out in der Weiterbildung vor. Der Begriff „Passung“ ist zudem zentral in ihrem Modell, welches situative Passungseinschätzungen als Verkettung von Bedingungen, Einschätzungen und Handlungen zu zeigen sucht. Dadurch wird das Prozesshafte von Passung sichtbar und Passungseinschätzungen werden prospektiv, situativ und retrospektiv in ihrer rahmenden Konstellation deutbar. Nicht-mehr-Teilnahme verstehen die Autorinnen und der Autor als Erweiterung eines binären Blicks der Teilnahmeforschung.

Den Einfluss von sozialer Einbettung und organisationalen Ressourcen auf die Lehrmotivation untersuchen *Uwe Wilkesmann und Ronja Vorberg*. Sie identifizieren eine Reihe von Fragen zu den spezifischen Voraussetzungen der Lehre in der wissenschaftlichen Weiterbildung in Deutschland und platzieren damit ihre Untersuchung in einem Feld, das Veränderungen in den hochschulpolitischen Rahmenbedingungen seit längerem anmahnt. Die Untersuchung nutzt einen umfangreichen Datensatz von 549 befragten Lehrenden in der wissenschaftlichen Weiterbildung und greift zur Analyse auf die Self-Determination-Theory zurück. Mittels einer Regressionsanalyse kann gezeigt werden, welche Faktoren Einfluss auf die Lehrmotivation der an einer Hochschule in der Weiterbildung Tätigen haben.

Mit Lehrkräften befasst sich auch der Beitrag eines Autorinnenkollektivs aus *Esther Winther, Jessica Paeßens, Beifang Ma, Monika Tröster und Beate Bowien-Jansen*. Lehrende in der Grundbildung weisen einen erheblichen Professionalisierungsbedarf auf, so die Autorinnen. Kollaboration verstehen sie als ein wichtiges Element der Lehrkräfteprofesisonalisierung und konstruieren in ihrer Evaluationsstudie ein Untersuchungssetting „kollaborativer Lernspielvalidierung“ für die finanzielle Grundbildung.

*Nicolas Echarti, Elisabeth Reichart und Pia Gerhards* geht es in ihrem Beitrag um die indikatorenbasierte Darstellung der Wirkungen beruflicher Weiterbildung. Diese möchten sie an die internationalen Vorgaben zur kontinuierlichen Bildungsberichterstattung anschlussfähig machen. Die Erfassung der Teilbereiche beruflicher Weiterbildung steht im Kontext der von der Bundesregierung verabschiedeten Nationalen Weiterbildungsstrategie und ist auch deshalb von Bedeutung, weil man eine Wirkung der Wirkungserfassung annehmen kann.

Erste Befunde aus dem DIE-Weiterbildungskataster stellen *Josef Schrader und Andreas Martin* vor. Das Kataster hat den Anspruch einer vollständigen und aktuellen Bestandsaufnahme der Weiterbildungsorganisationen in Deutschland und bietet damit vielfältige Potenziale sowohl für die Weiterbildungspolitik als auch für die Forschung. Der Beitrag ordnet das Weiterbildungskataster in die bestehende Forschung zur Weiterbildung ein, schildert die theoretischen Herausforderungen und das methodische Vorgehen bei der Datensammlung, Verifikation und Hochrechnung und präsentiert erste empirische Ergebnisse zur regionalen Verteilung.

Den Autorinnen und Autoren sowie Gutachterinnen und Gutachern möchten wir abschließend unseren Dank für ihre Beiträge zu diesem Themenheft der *Zeitschrift für Weiterbildungsforschung* aussprechen und wünschen den Lesenden eine anregende Lektüre.

