# Erratum zu: Erwachsenen- und Weiterbildung unter Pandemiebedingungen. Herausforderungen und Perspektiven

**DOI:** 10.1007/s40955-021-00199-y

**Published:** 2022-01-21

**Authors:** Kerstin Hoenig, Gabriele Molzberger

**Affiliations:** 1 Deutsches Institut für Erwachsenenbildung – Leibniz-Zentrum für Lebenslanges Lernen, Bonn, Deutschland; 2 Bergische Universität Wuppertal, Wuppertal, Deutschland

**Erratum zu:**

**ZfW 2021**

10.1007/s40955-021-00197-0

Im Editorial dieser Ausgabe wurde der Name der Koautorin des Beitrags „COVID-19 und Weiterbildung – Überblick zu Forschungsbefunden und Desiderate“ nicht erwähnt: Anika Denninger. Das Editorial wurde korrigiert und nochmals veröffentlicht. Wir bedauern diesen Fehler.

Der Originalbeitrag wurde korrigiert.

